# How Sleep Affects Daytime Functioning: The Latest Insights from Different Patient and Age Groups

**DOI:** 10.3390/brainsci11091163

**Published:** 2021-08-31

**Authors:** Ellemarije Altena, Jason G. Ellis

**Affiliations:** 1 Institut de Neurosciences Cognitives et Intégratives d’Aquitaine-UMR 5287 CNRS, Team Neuroimaging and Human Cognition, Université de Bordeaux, Zone Nord Bat 2, 2ème étage, 146 Rue Léo Saignat, CEDEX, 33076 Bordeaux, France; 2 Northumbria Sleep Research, Faculty of Health and Life Sciences, Northumbria University, 134 Northumberland Building, Newcastle upon Tyne NE1 8ST, UK; jason.ellis@northumbria.ac.uk

Sleep problems can have a major impact on daytime functioning across all domains (i.e., cognitive, affective and physical). It can affect attention, concentration and higher cognitive functions, such as working memory [1], as well as the levels of stress, anxiety and depression [2]. The prevalence of sleep problems has increased exponentially since the COVID-19 outbreak [3,4,5], underlining the urgency to prevent and treat sleep problems, in order to preserve long-term physical and mental health [6].

In this special issue, we address the important topic of sleep-related daytime functioning with publications on a variety of sleep disorders, their pathophysiological mechanisms, comorbid conditions and daytime consequences. First, the paper by Reddy and Van der Werf sheds light on the important function of sleep, during which replenishing and cleansing of the brain occurs by enhancing glymphatic clearance. Toxic effects on brain functioning that occur in the context of, for example, Alzheimer’s disease, can be limited by this clearance. The paper summarizes how lifestyle choices during the day and night, such as stress reduction and Omega-3 consumption, can further enhance this glymphatic clearance [7].

More sleep-related neural mechanisms are addressed in the manuscript by Gool et al. [8], in which they show that narcolepsy patients, who suffer from uncontrollable sleep fits during daytime, have problems performing attention tasks in a dose response manner with increasing complexity. This is accompanied by a lower activity in those brain regions important for stabilizing attention levels and inhibiting error-related motor activity, suggestive of affected executive functioning in narcolepsy patients [8].

Affected sustained attention is also reported by Angelelli et al., who investigated those with obstructive sleep apnea (OSA-breathing difficulties during the night), in relation to attention problems and sleepiness during daytime. Interestingly, OSA patients were shown to have difficulties in specific aspects of attention processes. The authors emphasize that measuring reaction time alone does not suffice in describing the variety of these problems, which include selective attention and performance stability [9].

The manuscript by Hill et al. then shows that apnea treatment in adults with Down syndrome, not only reduced sleepiness, but also resulted in reductions in anxiety and depression, while intelligence and global health scores also improved [10]. The findings by Angelelli et al. and Hull et al. underline the importance of investigating and treating sleep apnea thoroughly, in order to improve daytime functioning, particularly in vulnerable populations.

Focusing on a different sleep disorder, namely insomnia, the work by Bickly et al. proposes a new instrument to measure how this condition affects different types of daytime activities, such as work, care for children and physical exercise. The researchers show that while insomnia patients participate in most daytime activities, they report that it takes them more effort and that sleep affects their participation negatively [11], which warrants timely intervention for this chronic limiting condition.

The manuscripts by Kölbel et al., Bacaro et al. and Mughal et al. address different aspects of the vital topic of sleep problems in children. In young children from six months to three years, Bacaro et al. show that daytime naps and night-time sleep duration are lower than formal health guidelines suggest. These guidelines stipulate that period of time before falling asleep is inversely related to positive emotions, and that daytime naps play an important role in emotional regulation in toddlers [12]. Kölbel et al. show that different sleep disorders are prevalent in children and young adults suffering from sickle cell anemia (4–23 years of age), causing daytime sleepiness [13]. By comparing groups of 6-to-12-year olds with, and without, autism or fetal alcohol syndrome, Mughal et al. show that in all participants, cognitive performance was related to sleep quality, and that particularly those children with autism or fetal alcohol syndrome suffered from multiple sleep problems [14].

In summary, the importance of investigating daytime consequences of sleep problems is emphasized by a collection of excellent manuscripts highlighting different sleep disorders, how they affect daytime cognitive functioning and sleepiness, how they are affected in vulnerable populations and what pathophysiological mechanism underlie them. These reports warrant future research on the effects of sleep treatment on cognitive, affective and physical daytime functions, as well as global health.

## References

[1] Killgore W.D.S. (2010). Effects of sleep deprivation on cognition. Prog. Brain Res..

[2] Baglioni C., Spiegelhalder K., Lombardo C., Riemann D. (2010). Sleep and emotions: A focus on insomnia. Sleep Med. Rev..

[3] Jahrami H., Bahammam A.S., Bragazzi N.L., Saif Z., Faris M., Vitiello M.V. (2021). Sleep problems during the COVID-19 pandemic by population: A systematic review and meta-analysis. J. Clin. Sleep Med..

[4] Altena E., Baglioni C., Espie C.A., Ellis J., Gavriloff D., Holzinger B., Schlarb A., Frase L., Jernelöv S., Riemann D. (2020). Dealing with sleep problems during home confinement due to the COVID-19 outbreak: Practical recommendations from a task force of the European CBT-I Academy. J. Sleep Res..

[5] Pérez-Carbonell L., Meurling I.J., Wassermann D., Gnoni V., Leschziner G., Weighall A., Ellis J., Durrant S., Hare A., Steier J. (2020). Impact of the novel coronavirus (COVID-19) pandemic on sleep. J. Thorac. Dis..

[6] Cheng P., Casement M.D., Kalmbach D.A., Castelan A.C., Drake C.L. (2021). Digital cognitive behavioral therapy for insomnia promotes later health resilience during the coronavirus disease 19 (COVID-19) pandemic. Sleep.

[7] Reddy O.C., Van der Werf Y.D. (2020). The sleeping brain: Harnessing the power of the glymphatic system through lifestyle choices. Brain Sci..

[8] Gool J.K., Van Der Werf Y.D., Lammers G.J., Fronczek R. (2020). The Sustained Attention to Response Task Shows Lower Cingulo-Opercular and Frontoparietal Activity in People with Narcolepsy Type 1: An fMRI Study on the Neural Regulation of Attention. Brain Sci..

[9] Angelelli P., Macchitella L., Toraldo D.M., Abbate E., Marinelli C.V., Arigliani M., De Benedetto M. (2020). The neuropsychological profile of attention deficits of patients with obstructive sleep apnea: An update on the daytime attentional impairment. Brain Sci..

[10] Hill E.A., Fairley D.M., Williams L.J., Spanò G., Cooper S.-A., Riha R.L. (2020). Prospective Trial of CPAP in Community-Dwelling Adults with Down Syndrome and Obstructive Sleep Apnea Syndrome. Brain Sci..

[11] Bickley K., Lovato N., Lack L. (2021). The Sleep Impact on Activity Diary (SIAD): A Novel Assessment of Daytime Functioning in Insomnia. Brain Sci..

[12] Bacaro V., Feige B., Benz F., Johann A.F., De Bartolo P., Devoto A., Lombardo C., Riemann D., Baglioni C. (2020). The Association between Diurnal Sleep Patterns and Emotions in Infants and Toddlers Attending Nursery. Brain Sci..

[13] Kölbel M., Kirkham F.J., Dimitriou D. (2020). Developmental Profile of Sleep and Its Potential Impact on Daytime Functioning from Childhood to Adulthood in Sickle Cell Anaemia. Brain Sci..

[14] Mughal R., Hill C.M., Joyce A., Dimitriou D. (2020). Sleep and Cognition in Children with Fetal Alcohol Spectrum Disorders (FASD) and Children with Autism Spectrum Disorders (ASD). Brain Sci..

